# A novel method for calculating mean erythrocyte age using erythrocyte creatine

**DOI:** 10.18632/aging.103193

**Published:** 2020-05-11

**Authors:** Masashi Kameyama, Masafumi Koga, Toshika Okumiya

**Affiliations:** 1 Department of Diagnostic Radiology, Tokyo Metropolitan Geriatric Hospital and Institute of Gerontology, Tokyo, Japan; 2 Department of Internal Medicine, Hakuhokai Central Hospital, Hyogo, Japan; 3 Department of Biomedical Laboratory Sciences, Faculty of Health Sciences, Kumamoto University, Kumamoto, Japan

**Keywords:** creatine, erythrocyte, average RBC age, lifespan, hemolysis

## Abstract

Estimating the lifespan of erythrocytes is useful for the differential diagnosis of anemia. However, measuring the lifespan of erythrocytes was very difficult; therefore, it was seldom measured. Erythrocyte creatine (EC) decreases reflecting erythrocyte age. We developed a method to obtain mean erythrocyte age (*M_RBC_*) from *EC*.

We reanalyzed the previously published data from 21 patients with hemolytic anemia, which included *EC* and the half-life of ^51^Cr.

*M_RBC_* and log_*e*_ EC showed excellent significant linearity (*r* = −0.9475, *p* < 0.001), proving that it could be treated as a mono-exponential relationship within the studied range (*EC*: 1.45 – 11.76 μmol/g Hb). We established an equation to obtain *M_RBC_* (days) from *EC* (μmol/g Hb): *M_RBC_* = −22.84log_*e*_ EC + 65.83.

This equation allowed calculation of *M_RBC_* based on EC which has practical applications such as the diagnosis of anemia.

## INTRODUCTION

Estimating the lifespan of erythrocytes is useful for the differential diagnosis of anemia, as it is known that the erythrocyte lifespan in hemolytic patients is shortened [1]. Previously, obtaining the lifespan or mean age of erythrocytes was very difficult; therefore, it was seldom measured. Furthermore, supply of ^51^Cr, which is needed for measuring erythrocyte lifespan, was ceased in Japan in 2015 due to low demand. This left Japanese doctors unable to measure the erythrocyte lifespan of patients by means of ^51^Cr. Biotin-labeling [2, 3] is also used to measure the erythrocyte lifespan, however its procedure is very laborious as well, requiring aseptic labeling of the erythrocytes and repeated blood samplings. Breath carbon monoxide (CO) measurement [4, 5] also may be useful to estimate erythrocyte turnover; however, this technique cannot be applied to smokers. We have proposed a method to estimate erythrocyte mean age from HbA1c and average glucose [6]. However, the method needed a glycation constant to be determined by another method. Some indices such as reticulocyte and haptogloblin were not sensitive enough to indicate mild hemolysis. Cases with latent hemolysis were reported which showed normal reticulocyte and normal haptogloblin levels, and yet, they showed shortened erythrocyte lifespan [7–9].

Creatine in the cells is maintained by creatine transporters. Deficiency in these transporters leads to symptoms [10, 11]. Young erythrocytes have adequate transporter activity resulting in intracellular creatine being tens of times higher than in plasma. However, the activity of the transporter gradually diminishes, so that old erythrocytes cannot maintain this concentration gradient.

Erythrocyte creatine (EC) has been demonstrated to be an excellent indicator of hemolysis [12, 13]. Estimation of mean erythrocyte age (*M_RBC_*) using EC would be more convenient than the ^51^Cr method, as it requires only one blood sample. Though an increase in EC value has been correlated with shorter lifespan of erythrocytes, EC value itself has not previously been used for the estimation of *M_RBC_* directly. An estimation of *M_RBC_* would be more useful for quantitative assessment of patients than simple EC value. Moreover, *M_RBC_* derived by EC may be comparable with *M_RBC_* derived by other methods.

In this study, we aimed to formulate an equation to obtain *M_RBC_* from EC concentration based on a model.

## RESULTS

### Relationship between *M_RBC_* and log_*e*_ EC

**Figure 1:**
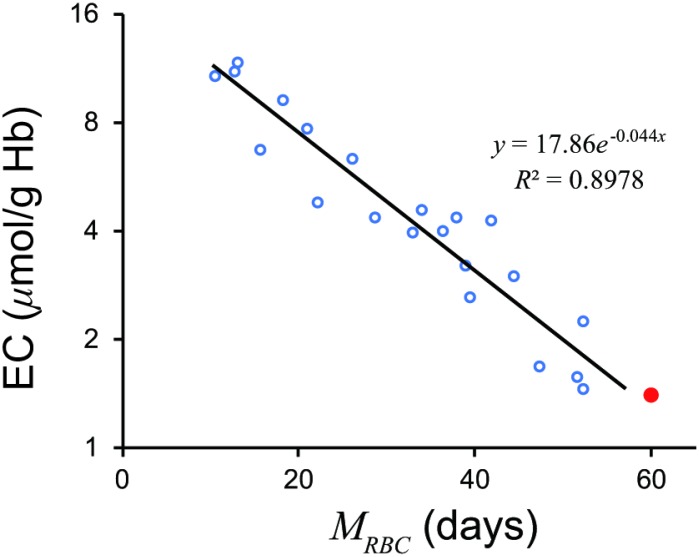
**Relationship between *M_RBC_* and log_*e*_ EC.** A significant linear relationship was observed. A *red closed circle* denotes a standard value; *M_RBC_* = 60 days, *EC* = 1.4μmol/g Hb. A *black line* denotes a regression line. *EC*, erythrocyte creatine; *M_RBC_*, mean erythrocyte age.

A significant linear relationship (*r* = −0.9475, df =19, *t* = 12.92, *p* = 7.368 × 10^−11^) was observed between ^51^Cr-derived *M_RBC_* and log*_e_ EC* (Figure 1). The relationship appears to be mono-exponential which is concurrent with the prediction by our model (Supplement) that the relationship would be bi- or mono-exponential.

A regression line was as follows.

$$\text{l}\text{o}{\text{g}}_{e}EC=-0.04379M_{RBC}+2.882$$ (1)

$$\Leftrightarrow M_{RBC}=-22.84\text{l}\text{o}{\text{g}}_{e}EC+65.83$$ (2)

A standard value of *EC* of 1.4 μmol/g Hb gives an *M_RBC_* of 58.14 days.

**Figure 2:**
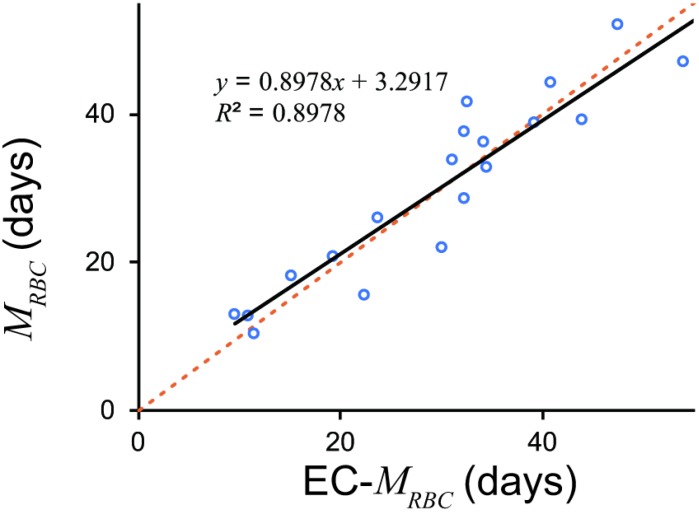
***M_RBC_* estimated by *EC* and ^51^Cr.** EC derived *M_RBC_* showed excellent estimation. An *orange dotted line* denotes line of identification (*y* = *x*). A *black line* denotes a regression line. *EC*, erythrocyte creatine; *M_RBC_*, mean erythrocyte age.

Equation (2) accurately estimated *M_RBC_* from *EC* values (Figure 2).

## DISCUSSION

The current study successfully established a reliable method of estimating *M_RBC_* from *EC* based on a creatine model (Supplement). We would be able to determine a glycation constant for the method to estimate erythrocyte mean age from HbA1c and average glucose [14].

**Figure 3:**
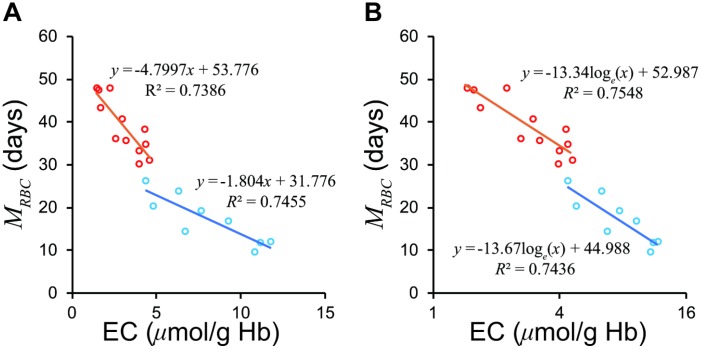
**Relationship between *EC* and *M_RBC_* in the groups with severe and mild hemolytic disease.** (**A**) The two groups show differing regression lines on a normal scale. (**B**) The two groups are unified on a semi-logarithmic scale. The *Red circles* represent mild group, *sky blue* the severe group according to Fehr et al. [13]. *EC*, erythrocyte creatine; *M_RBC_*, mean erythrocyte age.

Although Fehr et al. [13] divided patients into a severe hemolytic disease group and a group with milder forms of hemolysis, our model suggested that logarithm of *EC* may combine the two groups (Figure 3). The regression formula passed close to a standard value of *EC*, 1.4 μmol/g Hb and 60 days of *M_RBC_*, which proves the validity of the formula.

It cannot be determined which wing of the two lines (Supplement) the observed line of the log_*e*_ EC– *M_RBC_* relationship is on; *i.e.* whether the slope of the graph represents the rate constant for creatine diffusion (λ_1_) or the rate constant for decline in creatine transporter (λ_2_). Another equation may need to be developed for value ranges not explored in this study.

The devised method was formulated entirely based on the previously presented data from only 21 patients.

This method should be verified by further study with various hematological diseases including thalassemia and hereditary spherocytosis. Estimation of *M_RBC_* from ^51^Cr half-life may not be optimal, although we believe that it would be tolerable. The EC transporter activity function, *Be*^−λ2*t*^ relies solely on the assumption that the number of transporters reduces overtime randomly due to erythrocytes’ lack of nucleus. However, the linear relationship between log_*e*_ EC and *M_RBC_* confirms the assumption. The EC measuring method of Fehr et al. [13] used a diacetyl-*l*-naphthol chemical reaction, which is less sensitive than the recently developed *N*-methylcarbamoyl derivative of methylene blue, 10-*N*-methylcarbamoyl-3,7-*bis*(dimethylamino)phenothiazine (MCDP) enzyme method [15]. Further study on the validity of our proposed formula would be best done in a country where ^51^Cr is available.

## CONCLUSIONS

Our equation does allow calculation of *M_RBC_* based on EC, which has practical applications such as the diagnosis of anemia.

## MATERIALS AND METHODS

### Patients

Data from 21 patients with hemolytic anemia, that was published by Fehr et al. [13], was examined. As this is a re-analysis study, approval by the institutional review board was not required.

### Data conversion

We estimated *M_RBC_* by multiplying the half life of ^51^Cr by 2.61. As human erythrocytes do not obey the Poisson process [16], the term “half-life” is not entirely suitable for erythrocytes. Fehr et al. [13] determined ^51^Cr half-life, the elution-corrected ^51^Cr half-life, and the mean cell lifespan. The mean cell lifespan was not recorded in their table. The elution-corrected ^51^Cr half-life would provide an estimate of *M_RBC_*, considering that normal erythrocytes in a human have a similar lifespan [16]. However, their elution-corrected ^51^Cr half-life seems less concordant with EC rank. Complicated procedures sometimes reduce the stability of the system. Therefore, we chose the simple uncorrected ^51^Cr half-life in the same way as Fehr et al. [13]. Considering that *M_RBC_* for normal erythrocytes is about 60 days, and the normal range of ^51^Cr half-life was 23 – 27 days, multiplying ^51^Cr half-life by 2.61 (= 60/23) provides a good estimation of *M_RBC_* in practice.

The units for erythrocyte creatine concentration used by Fehr et al. [13] were mg/dL of red cells. We converted these into μmol/g Hb by the following equation, assuming mean cell hemoglobin concentration (MCHC) is 33g/dL. The molecular weight of creatine is 131.15. While MCHC varies naturally and decreases in iron deficiency anemia, variability in MCHC is generally low.

$$x\text{mg/dL}\;=\;x\;\times \;\frac{10^{3}}{131.15\;\times \;33}\mu \text{mol/g}\;\text{Hb}\;=\;\frac{x}{4.328}\mu \text{mol/g}\;\text{Hb}$$ (3)

### Data analysis

Data on *EC* and *M_RBC_* were analyzed with a spreadsheet software, Excel ^® ^ 365 (Microsoft Corporation, Redmond, WA, USA).

Logarithms of *EC* and *M_RBC_* were plotted based on our model (Supplement).

## Supplementary Material

Supplementary Materials
