# Supplementary material for: A novel method for calculating mean erythrocyte age using erythrocyte creatine
Source: Aging (Albany NY). 2020 May 11;12(9):8702–9. doi: 10.18632/aging.103193 (PMC7244067; doi:10.18632/aging.103193)
Supplement: Supplementary Materials [file aging-12-103193-s001..pdf]

SUPPLEMENTARY MATERIALS

Supplementary Methods

Theory

The terms in the equations are summarized in Table 1.

Creatine in a single erythrocyte

The rate of diffusion of creatine would be proportional to the concentration of creatine in the cell. We assume that the transporter activity obeys an exponential function; *i.e.* transporters diminish randomly (proportional to the number of the transporter) and are not renewed due to lack of nucleus.

$$\frac{dCr}{dt} = -\lambda_1 Cr(t) + Be^{-\lambda_2 t} \qquad (1)$$

where $Cr(t)$ denotes creatine concentration in a t-day-old erythrocyte, λ_1 denotes rate constant of creatine diffusion. $Be^{-\lambda_2 t}$ ($B > 0$) is creatine transporter activity. This differential equation can be solved analytically if $\lambda_1 \neq \lambda_2$.

$$\frac{d(Cr - \frac{B}{\lambda_1 - \lambda_2} e^{-\lambda_2 t})}{dt} = -\lambda_1 Cr(t) + \frac{B\lambda_1}{\lambda_1 - \lambda_2} e^{-\lambda_2 t} \qquad (2)$$

$$Cr(t) = Ae^{-\lambda_1 t} + \frac{B}{\lambda_1 - \lambda_2} e^{-\lambda_2 t} \qquad (3)$$

where A denotes an integral constant.

$Cr(t)$ is a sum of two exponential functions, it can be treated as a bi-exponential function or a monoexponential function within a range of interest.

The fate of $Cr(t)$ is dependent on whether $\lambda_1 - \lambda_2 > 0$ or not. If $\lambda_1 - \lambda_2 < 0$ and $C'(0) > 0$, $Cr(t)$ has a peak when $t > 0$ (Figure 1 *orange line*). It can be treated as a mono-exponential function after the second term is negligible. However, as EC monotonically and rapidly decreases after birth of the erythrocytes [1], we can exclude this condition.

If $\lambda_1 - \lambda_2 < 0$ and $C'(0) \leq 0$, $Cr(t)$ decreases monotonically. As $\lambda_2 > \lambda_1$, $e^{-\lambda_2 t}$ decreases more rapidly. Moreover, the second negative term must be small, considering that $C'(0) < 0$ yields $\frac{B}{\lambda_2 - \lambda_1} < \frac{\lambda_1}{\lambda_2} A$. Therefore, the second negative term can be considered negligible comparing the first term.

Table 1. Terms used in the text.

Term	Definition	Representative value
$Cr(t)$	creatine concentration in a t-day-old erythrocyte	
λ_1	rate constant for creatine diffusion	
λ_2	rate constant for decline in creatine transporter	
λ	substitute for λ_1 or λ_2	
A, B, C, D	constants	
EC	mean erythrocyte creatine concentration	1.4 µmol/g Hb
α	a parameter of gamma distribution	25.59
β	a parameter of gamma distribution	5.59
$p(t)$	probability density function of RBC death	
R_0	erythrocyte production rate	– /day
$R(t)$	the number of erythrocytes at t days after birth	
M_{RBC}	mean red blood cell age	60 days
RBC	number of erythrocytes	–

If $\lambda_1 - \lambda_2 > 0$, $Cr(t)$ is a bi-exponential function. The logarithm of a bi-exponential function can be expressed by a bent line (Figure 1B *blue line*), because a large t makes one term negligible, while small t ($\rightarrow -\infty$) makes the other term negligible.

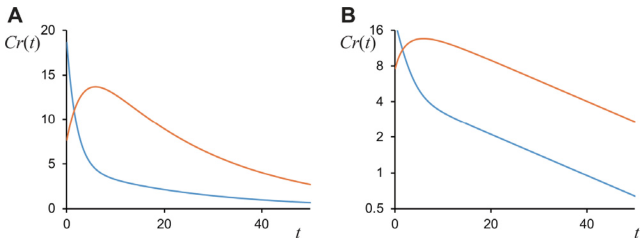

Figure 1. Examples of $Cr(t)$. (**A**) normal scale; (**B**) logarithmic scale. *Orange line:* $\lambda_1 - \lambda_2 < 0$ and $C'(0) > 0$, the function has a peak. *Blue line:* $\lambda_1 - \lambda_2 > 0$, $Cr(t)$ is a bi-exponential function. The logarithm of a bi-exponential function can be expressed by a bent line.

Problem applying single cell model to erythrocyte population

Equation (3) itself would not be suitable to obtain mean erythrocyte age, because EC is not measured from a single cell.

$$EC = \left(\sum_{i}^{n} Cr(t_i) \right) / n = A \left(\sum_{i}^{n} e^{-\lambda_1 t_i} \right) / n + \frac{B}{\lambda_1 - \lambda_2} \left(\sum_{i}^{n} e^{-\lambda_2 t_i} \right) / n \quad (4)$$

$y = e^{-\lambda x}$ is downward convex. The centroid of n-polygonal, $(t_i, e^{-\lambda t_i})$ is over the curve of $y = e^{-\lambda x}$.

$$\left(\sum_{i}^{n} e^{-\lambda t_i} \right) / n \geq \exp\left(-\lambda \frac{\sum_{i}^{n} t_i}{n} \right) \quad (5)$$

Therefore, when $\lambda_1 - \lambda_2 > 0$,

$$EC \geq Ae^{-\lambda_1 M_{RBC}} + \frac{B}{\lambda_1 - \lambda_2} e^{-\lambda_2 M_{RBC}} \quad (6)$$

Erythrocyte lifespan

Kameyama et al. [2] have recently calculated RBC lifespan based on the probability density function $p(t)$ of RBC death proposed by Shrestha et al. [3].

$$p(t) = \frac{1}{\Gamma(\alpha)\beta^{\alpha}} t^{\alpha-1} e^{-t/\beta} \quad (7)$$

Γ denotes the Euler gamma function.

$$\Gamma(\alpha) = \int_0^{\infty} x^{\alpha-1} e^{-x} dx \quad (8)$$

The number of erythrocytes (RBC) and mean erythrocyte age (M_{RBC}) was calculated. (See Kameyama et al. [2] for details.)

$$RBC = R_0 \int_0^{\infty} tp(t)dt = R_0 \alpha\beta \quad (9)$$

$$M_{RBC} = \frac{(\alpha+1)\beta}{2} \quad (10)$$

Creatine model

The number of t-day-old erythrocyte is $R(t)$. Each RBC has $Ae^{-\lambda_1 t} + \frac{B}{\lambda_1 - \lambda_2} e^{-\lambda_2 t}$ creatine. Therefore, mean creatine concentration, EC can be described as follows:

$$EC = \int_0^{\infty} R(t) \times \left(Ae^{-\lambda_1 t} + \frac{B}{\lambda_1 - \lambda_2} e^{-\lambda_2 t} \right) dt / RBC \quad (11)$$

$$\int_0^{\infty} R(t) \times e^{-\lambda t} dt = \left[R(t) \frac{e^{-\lambda t}}{-\lambda} \right]_0^{\infty} - \int_0^{\infty} R'(t) \frac{e^{-\lambda t}}{-\lambda} dt = \frac{R_0}{\lambda} - \frac{R_0}{\lambda} \int_0^{\infty} p(t) e^{-\lambda t} dt \quad (12)$$

$$\int_0^{\infty} p(t) e^{-\lambda t} dt = \frac{1}{\Gamma(\alpha)\beta^{\alpha}} \int_0^{\infty} t^{\alpha-1} e^{-(1/\beta+\lambda)t} dt = \frac{1}{\Gamma(\alpha)\beta^{\alpha}} \frac{\Gamma(\alpha)}{(1/\beta+\lambda)^{\alpha}} = \frac{1}{(1+\beta\lambda)^{\alpha}} \quad (13)$$

Hence, EC can be expressed as follows:

$$EC = \frac{A}{\lambda_1 \alpha\beta} \left(1 - \frac{1}{(1+\beta\lambda_1)^{\alpha}} \right) + \frac{B}{\lambda_1 - \lambda_2} \frac{1}{\lambda_2 \alpha\beta} \left(1 - \frac{1}{(1+\beta\lambda_2)^{\alpha}} \right) \quad (14)$$

Approximation of the derived relationship

The Taylor expansion provides the following equation:

$$(1+\beta\lambda)^{-\alpha} \approx 1 - \alpha\beta\lambda + \frac{\alpha(\alpha+1)}{2}(\beta\lambda)^2 - \frac{\alpha(\alpha+1)(\alpha+2)}{6}(\beta\lambda)^3 + \ldots \quad (15)$$

$$\frac{1}{\lambda\alpha\beta}\left(1 - \frac{1}{(1+\beta\lambda)^{\alpha}}\right) \approx \frac{1}{\lambda\alpha\beta}\left(\alpha\beta\lambda - \frac{\alpha(\alpha+1)}{2}(\beta\lambda)^2 + \frac{\alpha(\alpha+1)(\alpha+2)}{6}(\beta\lambda)^3 - \ldots\right) = 1 - \frac{\beta(\alpha+1)}{2}\lambda + \frac{(\alpha+1)(\alpha+2)}{6}(\beta\lambda)^2 - \ldots \quad (16)$$

As $\alpha >> 1$,

$$\frac{1}{\lambda\alpha\beta}\left(1 - \frac{1}{(1+\beta\lambda)^{\alpha}}\right) \approx 1 - \lambda M_{RBC} + \frac{2}{3}\lambda^2 M_{RBC}^2 - \ldots \quad (17)$$

Thus, $\frac{1}{\lambda\alpha\beta}\left(1-\frac{1}{(1+\beta\lambda)^{\alpha}}\right)$ can be described approximately as a function of M_{RBC}. Although how α and β vary when M_{RBC} decreases or increases cannot be determined, this implies that the function $\frac{1}{\lambda\alpha\beta}\left(1-\frac{1}{(1+\beta\lambda)^{\alpha}}\right)$ would not be greatly affected by α and β if $M_{RBC}=(\alpha+1)\beta/2$ is satisfied. This can be confirmed numerically (Figure 2A). Calculations based on the assumption that β was constant and β was proportionate to α showed similar results. Therefore, β can be considered a constant.

Below, $\frac{1-e^{-x}}{x}$ was approximated to be De^{-Cx}, as the shape of the graphs were similar (Figure 2B).

$$C=\frac{-x_0e^{-x_0}+(1-e^{-x_0})}{x_0(1-e^{-x_0})},\qquad D=\frac{1-e^{-x_0}}{x_0e^{-Cx_0}} \qquad (18)$$

provides the same value of the two function and the differential function at $x=x_0$. Figure 2B visualizes the approximation.

As $\frac{1-e^{-x}}{x}$ can be treated as an exponential function, it follows that $\frac{1}{\lambda\beta x}\left(1-\frac{1}{(1+\beta\lambda)^{x}}\right)$ can also be treated as an exponential function, because

$$\begin{aligned}&\frac{1}{\lambda\beta\alpha}\left(1-\frac{1}{(1+\beta\lambda)^{\alpha}}\right)\\&=\frac{\log_e(1+\beta\lambda)}{\lambda\beta}\frac{1-\exp(-\log_e(1+\beta\lambda)\alpha)}{\log_e(1+\beta\lambda)\alpha}\\&\approx\frac{\log_e(1+\beta\lambda)}{\lambda\beta}D\exp\left(-C\log_e(1+\beta\lambda)\left(\frac{2M_{RBC}}{\beta}-1\right)\right)\end{aligned} \qquad (19)$$

C, D can be estimated by equation (18). To obtain an approximation around M_{RBC0}, x_0 should be as following:

$$x_0=\log_e(1+\beta\lambda)\left(\frac{2M_{RBC0}}{\beta}-1\right) \qquad (20)$$

In conclusion, EC can be expressed approximately as a (bi-)exponential function of M_{RBC}.

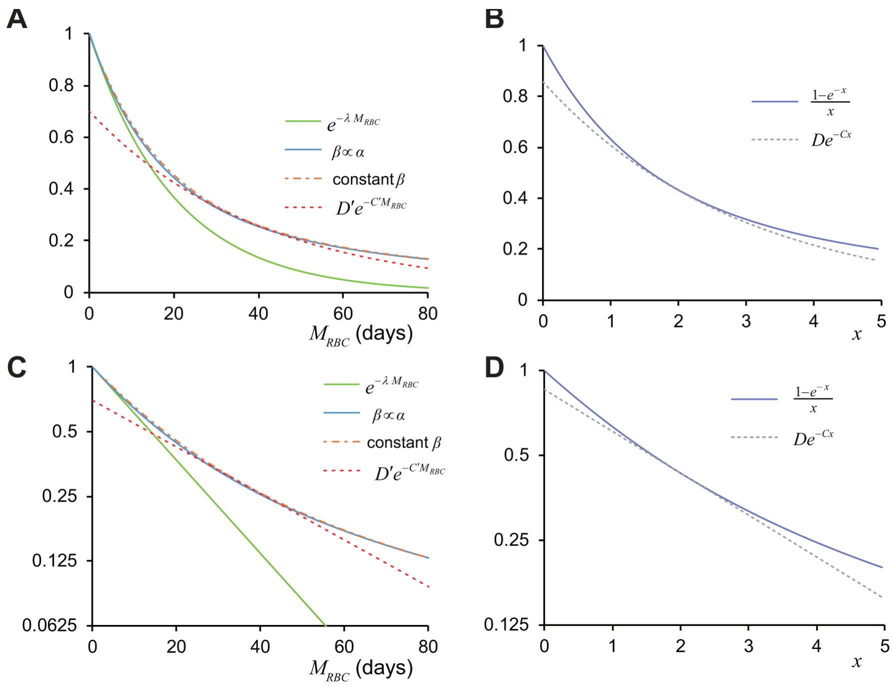

Figure 2. (**A**) Relatiohship between M_{RBC} and $\frac{1}{\lambda\alpha\beta}\left(1-\frac{1}{(1+\beta\lambda)^{\alpha}}\right)$. The two condition of $\frac{1}{\lambda\alpha\beta}\left(1-\frac{1}{(1+\beta\lambda)^{\alpha}}\right)$ (constant β and $\beta\propto\alpha$) showed similar results. Note that $\frac{1}{\lambda\alpha\beta}\left(1-\frac{1}{(1+\beta\lambda)^{\alpha}}\right)$ is consistently larger than $e^{-\lambda M_{RBC}}$. $D'e^{-C'M_{RBC}}$ is an approximation at $M_{RBC}=36.33$ with exponential function by equation (19). (**B**) $\frac{1-e^{-x}}{x}$ and its approximation, De^{-Cx} (equation (18)) when $x_0=2$. (**C**, **D**) Semi-log scales of (**A**, **B**) show that $\frac{1-e^{-x}}{x}$ and $\frac{1}{\lambda\alpha\beta}\left(1-\frac{1}{(1+\beta\lambda)^{\alpha}}\right)$ can be treated as an exponential function.

Supplementary References

1. Okumiya T, Kageoka T, Hashimoto E, Park K, Sasaki M. [Clinical usefulness of measurement of creatine contents in human erythrocytes as an index of erythropoiesis]. Rinsho Byori. 1992; 40:165–71. PMID:1583789

2. Kameyama M, Takeuchi S, Ishii S. Steady-state relationship between average glucose, HbA1c and RBC lifespan. J Theor Biol. 2018; 447:111–17. https://doi.org/10.1016/j.jtbi.2018.03.023 PMID:29559230

3. Shrestha RP, Horowitz J, Hollot CV, Germain MJ, Widness JA, Mock DM, Veng-Pedersen P, Chait Y. Models for the red blood cell lifespan. J Pharmacokinet Pharmacodyn. 2016; 43:259–74. https://doi.org/10.1007/s10928-016-9470-4 PMID:27039311
